# Patients seeking stem cell therapies—a prospective qualitative analysis from a Regenerative Medicine Consult Service

**DOI:** 10.1038/s41536-022-00215-w

**Published:** 2022-03-25

**Authors:** Jennifer R. Arthurs, Lisa M. Nordan, Brian H. Hultgren, Michael G. Heckman, Dayana Martinez, Zubin Master, Shane A. Shapiro

**Affiliations:** 1 Center for Regenerative Medicine, Mayo Clinic, Jacksonville, FL USA; 2 Robert D. and Patricia E. Kern Center for the Science of Health Care Delivery, Mayo Clinic, Jacksonville, FL USA; 3 Division of Biomedical Statistics and Informatics, Mayo Clinic, Jacksonville, FL USA; 4 United States Navy, Washington, DC USA; 5 Biomedical Ethics Research Program and the Center for Regenerative Medicine, Mayo Clinic, Rochester, MN USA; 6 Department of Orthopedic Surgery, Mayo Clinic, Jacksonville, FL USA; 7 Present Address: Center for Regenerative Medicine, Mayo Clinic, Jacksonville, FL USA

**Keywords:** Translational research, Medical ethics

## Abstract

Despite patient demand for stem cell therapies (SCTs) for musculoskeletal conditions, there remains limited research on why patients seek SCTs or their sources of information. We employ three questions into a consult intake form: (1) Why are you interested in stem cell treatment for your condition? (2) How did you find out about stem cell treatment for your condition? (3) Have you contacted a stem cell clinic? Responses analyzed, using a qualitative content analysis approach to identify themes reveal many patients seek SCTs to treat pain or delay surgery which may align with some current clinical evidence while other patients express motivations as expected outcomes (e.g., SCTs are better than standard of care or can regenerate tissue) which are not supported by current medical evidence. These differences suggests that patient-centered counseling may help patients by addressing misconceptions and increasing health literacy about expected outcomes of SCTs for treating musculoskeletal conditions.

## Introduction

Orthobiologics are substances derived from the body that are used to reduce pain and aid in the repair of musculoskeletal diseases or injuries. One component of orthobiologic medicine becoming more prevalent in use over the last decade involves point of care interventions for musculoskeletal pain and injuries^1^. in which human cells are concentrated but not manipulated or added to other substances and subsequently reimplanted or reinjected back to the site of orthopedic injury in a regulatory compliant manner. Though robust supporting evidence is lacking for most conditions, emerging support for others has demonstrated the potential for orthobiologics to improve validated patient outcomes in orthopedic disease^2–4^. Perhaps as a result of such early progress, promises of regenerative medicine have once again become hyped, accompanied by misleading claims related to the clinical readiness of regenerative and stem cell interventions. This has contributed to inflated patient expectations and a skewed public understanding of such treatments^5^. Public demand for stem cell and regenerative interventions to some degree, have created a direct-to-consumer market with about 60% of these in the orthopedic space^6–9^. Furthermore, some orthobiologic options, like platelet rich plasma do not contain stem cells yet are captured within the “stem cell” treatment (SCT) market. The growth of orthobiologics has resulted in many orthopedic and sports medicine societies publishing statements and recommending the responsible use of cell-based interventions^10–13^. As a result, responsible practitioners have attempted to navigate the difficult task of appropriately translating orthobiologic therapies despite the absence of clear clinical evidence in some applications and the presence of confounding and misleading claims permeating the field.

Patients with unmet needs actively seek potential treatments for their condition from a range of sources. In addition to online website, blog and social media searches^5,14–18^, many patients seeking information about regenerative options also actively ask advice from friends, family, providers, and consultation services^19–22^. Among one of the most prolific regenerative medicine consult services in the U.S., nearly 60% of patients queried information and advice surrounding regenerative interventions for orthopedic conditions^19^. Several studies have highlighted the skewed marketing of the direct-to-consumer regenerative interventions which tends to overemphasize benefits, underestimate risks, and promote regenerative options over standard of care among^5,14,23^, including in the use of stem cell or orthobiologic therapies^24^. These studies suggest that some patients may be misinformed about the science, safety, and efficacy of novel orthobiologics, and may choose options that are not in their best health interest. Yet not all patients considering an unproven stem cell and regenerative intervention are duped by misinformation. Patients have reported that they are well-informed having conducted extensive research^18,25^ and know about the risks and have reasonable expectations about the benefits of unproven regenerative products (unpublished observations). This gets further complicated in orthobiologics where clinical studies of specific interventions for specific musculoskeletal conditions have been shown to be safe and may have some benefit^26^. Here, patients may consider a regenerative option as less invasive way to delay surgery or to provide temporary relief.

Despite the growing demand for orthobiologics by the public, little is known about why patients are considering these interventions, their expectations, and their sources of information^27^. Knowing sources of information and the reasons and values patients bring when deciding whether to undertake an orthobiologic or SCT is important to provide patients evidence-based information and counseling. This information may also help physicians reduce unrealistic optimism and taper expectations about orthobiologic and SCTs.

As part of the responsible advancement of regenerative medicine, we have developed a Regenerative Medicine Consult Service at Mayo Clinic to communicate specialized information^19^, and currently, our database has over a thousand patients contacting the Florida service alone for orthopedic considerations. This platform provides a unique opportunity to capture the views of patients considering orthobiologic options. In addition to patient-reported outcome measures, patients approaching the consult service provide health and treatment information related to their conditions which are captured in the Regenerative Evidence Based Outcomes registry^28^. As part of this intake procedure, we routinely capture the reasons why patients are considering stem cell and regenerative medicine interventions, their sources of information, and whether they have previously contacted a regenerative medicine clinic. In this paper, we explore patient-reported responses to these questions to identify a common set of themes. We reason that patients have a diverse set of reasons for seeking SCTs, some of which may be based on current clinical evidence surrounding orthopedic research.

## Results

### Characteristics of patients seeking SCTs

A total of 533 patient responses were analyzed. Median age was 68 years (range: 18–93 years) and 50% of patients were male. The majority of patients (64.7%, *n* = 345) came in for an in-person consultation while the remainder answered the intake questions but declined consultation.

### Reasons why patients seek SCTs

The most common reason patients reported being interested in a SCT for their condition(s) was avoiding or delaying joint replacement or tendon repair (27.4%, *n* = 146) followed by treating or alleviating pain (26.5%, *n* = 141). Nearly a fifth (18.9%, *n* = 101) reported wanting a SCT because it was either less invasive than surgery, better than surgery, or better than standard of care, which included steroid injections, medications, or physical therapy. Some patients provided responses in terms of the ability of stem cells to repair or regenerate tissue (8.4%, *n* = 45), restore function (7.9%, *n* = 42), or a preference to try an alternative treatment as nothing else had worked for them (5.3%, *n* = 28), or to try a natural option (1.1%, *n* = 6). These and additional responses are summarized in Table 1. Specific examples of quotes from patients are provided for each category in Supplementary Table 2.

**Table 1:** Reasons why patients reported that they were interested in a SCT for their condition.

Categories*	No. (%) of patients (*N* = 533)
Avoid/delay joint replacement or tendon repair	146 (27.4%)
Treat or alleviate pain	141 (26.5%)
Stem cells less invasive than surgery, better than surgery or better than standard of care (medications, steroid injections, physical therapy)	101 (18.9%)
Repair, regenerate tissue	45 (8.4%)
Restore function	42 (7.9%)
Try alternative option, because nothing else worked	28 (5.3%)
Referred by friend/family or friend/family had benefit from a regenerative medicine treatment	17 (3.2%)
Referred by another healthcare provider	13 (2.4%)
Previously had regenerative therapy and found it to be helpful	8 (1.5%)
Medical contraindication to surgery	6 (1.1%)
Try natural option	6 (1.1%)
Interested in research	4 (0.8%)
Other or unclear response	32 (6.0%)
Total	598

*Patients with multiple responses all included.

Table 2 compares the responses of patients who did attend an in-person consultation from those who did not. Patients who presented for in-person consultation more often responded that treating pain (30.1% vs. 19.7%, *P* = 0.010) was their primary reason for seeking regenerative therapy than those who did not want an in-person consultation. Additionally, patients who presented for in-person consultations less often responded that SCTs were less invasive/better than surgery, or better than standard of care (15.9% vs. 24.5%, *P* = 0.020). Finally, patients that presented for in-person consultations more commonly had a specific reason for the consultation and less commonly gave an unclear response (0.6% vs. 16.0%, *P* < 0.001) than those that declined in-person consultation.

**Table 2:** Comparison of reasons why patients reported that they were interested in a SCT for their condition according to whether they had an in-person consultation.

	In-person face to face consult		
Category	No (*N* = 188)	Yes (*N* = 345)	*P* value
Avoid/delay joint replacement or tendon repair	45 (23.9%)	101 (29.3%)	0.22
Treat or alleviate pain	37 (19.7%)	104 (30.1%)	0.010
Stem cells less invasive than surgery, better than surgery or better than standard of care (medications, steroid injections, physical therapy)	46 (24.5%)	55 (15.9%)	0.020
Repair, regenerate tissue	22 (11.7%)	23 (6.7%)	0.051
Restore function	11 (5.9%)	31 (9.0%)	0.24
Try alternative option, because nothing else worked	7 (3.7%)	21 (6.1%)	0.31
Referred by friend/family or friend/family had benefit from a regenerative medicine treatment	6 (3.2%)	11 (3.2%)	1.00
Referred by another healthcare provider	2 (1.1%)	11 (3.2%)	0.15
Previously had regenerative therapy and found it to be helpful	1 (0.5%)	7 (2.0%)	0.27
Medical contraindication to surgery	2 (1.1%)	4 (1.2%)	1.00
Try natural option	2 (1.1%)	4 (1.2%)	1.00
Interested in research	3 (1.6%)	1 (0.3%)	0.13
Other or unclear response	30 (16.0%)	2 (0.6%)	<0.001
*P* values result from Fisher’s exact test.			

### How patients find out about SCTs: information sources and interpersonal

We also queried patients to capture how they found out about SCTs in order to understand the sources of information they used when considering a regenerative option (Table 3). Most patients performed online research to find out about available SCTs for their condition (39.8%, *n* = 212), which was followed by interpersonal communication including recommendations from a friend or family member (19.9%, *n* = 106), or a healthcare provider referral (19.3%, *n* = 103). Sources of media which includes both social media and television stories (but not television advertisements) made up 9.2% (*n* = 49) of responses. Some patients (5.4%, *n* = 29) reported receiving information about stem cells from stem cell seminars and 3.2% of patients (*n* = 17) reported finding out about SCTs from television or print advertisements. Less than 1% of patients (*n* = 4) reported hearing about SCTs from ClinicalTrials.gov.

**Table 3:** Responses for how patients found out about a SCT for their condition.

Responses	No. (%) of patients (*N* = 576)
“Internet” Research or Online “Search”	212 (39.8%)
Recommended by friend/family	106 (19.9%)
Healthcare provider referral or existing Mayo Clinic patient	103 (19.3%)
Social Media, video or TV segment (non-advertisement), etc.,	49 (9.2%)
Mayo Clinic Story, Media or Communications	47 (8.8%)
Stem cell seminar or stem cell clinic	29 (5.4%)
Advertisement (TV or print)/non-internet	17 (3.2%)
Clinicaltrials.gov	4 (0.8%)
I am also scientist or healthcare provider	4 (0.8%)
Other or unclear response	5 (0.9%)

Patients who had presented for in-person consultation more often learned about the regenerative medicine through Mayo Clinic media (10.7% vs. 5.3%, *P* = 0.038) and through a provider referral (22.6% vs. 13.3%, *P* = 0.011), and less often through online research (36.5% vs. 45.7%, *P* = 0.042) or television/print advertisement/story (0.3% vs. 8.5%, *P* < 0.001). These calculations are summarized in Table 4.

**Table 4:** Comparison of responses for how patients found out about a SCT for their condition according to whether they had an in-person consultation.

	In-person consultation		
How the patient found out about a stem cell treatment for their condition	No (*N* = 188)	Yes (*N* = 345)	*P* value
“Internet” Research or Online “Search”	86 (45.7%)	126 (36.5%)	0.042
Recommended by friend/family	44 (23.4%)	62 (18.0%)	0.14
Healthcare provider referral or existing Mayo Clinic patient	25 (13.3%)	78 (22.6%)	0.011
Social Media, video or TV segment (non-advertisement), etc.,	9 (4.8%)	40 (11.6%)	0.011
Mayo Clinic Story, Media or Communications	10 (5.3%)	37 (10.7%)	0.038
Stem cell seminar or stem cell clinic	12 (6.4%)	17 (4.9%)	0.55
Advertisement (TV or print)/non-internet	16 (8.5%)	1 (0.3%)	<0.001
Clinicaltrials.gov	3 (1.6%)	1 (0.3%)	0.13
I am also scientist or healthcare provider	2 (1.1%)	2 (0.6%)	0.62
Other or unclear response	1 (0.5%)	3 (0.9%)	1.00
*P* values result from Fisher’s exact test.			

### Patients previous contact with stem cell clinics

Many patients had contacted stem cell clinics to speak with them about a SCT for their condition before approaching our consultation service (40%, *n* = 214). A comparison of reasons why patients reported that they were interested in a SCT and whether they had contacted a clinic are revealed no significant differences for the two groups for any of the reasons listed (all *P* ≥ 0.086). Despite this, patients who had an in-person consult were less likely to have contacted a clinic to speak to them about a SCT (37% vs. 47%, *P* = 0.026) Table 5.

**Table 5:** Comparison of reasons why patients reported that they were interested in a SCT for their condition according to whether they had contacted a clinic to speak to them about a SCT for their condition.

	Contacted a clinic to speak to them about a SCT for their condition		
Reason why interested in a SCT	No (*N* = 319)	Yes (*N* = 214)	*P* value
Avoid/delay joint replacement or tendon repair	82 (25.7%)	64 (29.9%)	0.32
Treat or alleviate pain	88 (27.6%)	53 (24.8%)	0.49
Stem cells less invasive than surgery, better than surgery or better than standard of care (medications, steroid injections, physical therapy)	58 (18.2%)	43 (20.1%)	0.58
Repair, regenerate tissue	24 (7.5%)	21 (9.8%)	0.43
Restore function	20 (6.3%)	22 (10.3%)	0.10
Try alternative option, because nothing else worked	19 (6.0%)	9 (4.2%)	0.43
Referred by friend/family or friend/family had benefit from regenerative medicine treatment	8 (2.5%)	9 (4.2%)	0.32
Referred by another healthcare provider	9 (2.8%)	4 (1.9%)	0.58
Previously had regenerative therapy and found it to be helpful	5 (1.6%)	3 (1.4%)	1.00
Medical contraindication to surgery	2 (0.6%)	4 (1.9%)	0.23
Try natural option	5 (1.6%)	1 (0.5%)	0.41
Interested in research	1 (0.3%)	3 (1.4%)	0.31
Other or unclear response	18 (5.6%)	14 (6.5%)	0.71
*P* values result from Fisher’s exact test.			

## Discussion

Understanding patients’ health knowledge and intentions when considering orthobiologics and SCTs is crucial in order to help patients navigate the various clinical options appropriate for their care needs. Our cohort of 533 patients and their prospective responses is the largest sampling of patient considerations for SCTs and provided valuable insight as to why patients are interested in SCTs, and their sources of information including whether they had contacted a clinic. We found that while many patients were driven by a reasonable desire to treat/alleviate pain and restore function (26.5% and 7.9% respectively), other patients expressed motivations not supported by existing medical evidence, such as the desire to avoid surgery, repair/regenerate torn tissue (8.4%), or that SCTs were superior to other standard of care treatments (up to 18.9%). Although it is beyond the scope of this study to delve into the clinical orthobiologics literature, there is no strong clinical evidence to suggest that joint replacement or tendon repair is fully avoidable, that SCTs are better than standard of care or surgical options, or that orthobiologics serve to regenerate the damaged tissues^10,26^. These results suggest that at least some portion of the patients contacting our consult service may be misinformed about efficacy or mechanism of action of regenerative options and how they can help treat musculoskeletal conditions.

Several studies show that clinic websites and social media have significant misinformation about regenerative medicine including the level of clinical evidence, safety, and efficacy^5,15–17,23,29,30^. It is widely known that patient text and video testimonials constitute a major source of “evidence” used to demonstrate efficacy to patients^5,23,30^, but it remains unknown when and how such testimonials are recorded and their veracity. One study among 59 stem cell businesses showed that only 7–8% of websites reported evidence based on registered or unregistered clinical trials and 35.6% reported data from scientific publications^30^. In another study, Kingery et al. examined websites of 896 clinic practices offering SCTs for musculoskeletal conditions and found that 96% had at least one piece of misinformation with an average of 4.6 statements of misinformation^24^. Our results about patients being misinformed is corroborated by a study which showed that patients considering, but not having yet undertaken, a SCT for osteoarthritis reported expecting an improvement in their condition and that the benefits outweighed the risks^27^. But even with the potential for inaccurate scientific information found on clinic websites, patients may not wholeheartedly believe what they read or act on it by undertaking an unproven intervention. In our ongoing interview studies with patients considering SCTs for a host of conditions, we found that patient decisions on whether to undergo an unproven intervention was complex and dependent on multiple factors including the severity of their condition, whether they are in a loss frame, considerations of medical risks, and trust in various actors and institutions. While knowledge and health literacy do play a role in health decision-making, it is unlikely to be the dominant factor guiding health behavior. As 40% of our patient cohort have undertaken online research about SCTs and 40% reported contacting a clinic, many patients are likely to have been exposed to potentially inaccurate information about orthobiologic and SCTs. Despite this, though our cohort may have been exposed to misinformation, it remains unclear the impact of such misinformation in shaping patient behavior to undertake an unproven orthobiologic or SCT.

Our results also showed that almost 65% of patients contacting our consult service attended the in-person consultation after completing our intake form. Patients that attended our in-person consultation were more likely to explain their primary reason for seeking a SCT was to treat pain and were less likely to report that SCTs were better than standard of care or surgery. Additionally, larger percentage of patients who had an in-person consultation were less likely to have contacted a clinic to speak to them about a SCT for their condition than patients who did not have an in-person visit. While we cannot know the exact reasons why some patients call for information about regenerative therapies then decline an in-person consultation, it is possible that the majority who attended in-person consultations had a stronger interest in receiving more information. Several studies show that patients believe they are already well-informed^18,21,22^ including one specifically reporting that patients seeking SCTs for musculoskeletal conditions were well informed by conducting their own research^27^. This may explain why patients declined the in-person consultations. Another possible reason for why patients did not seek out an in-person consult could be due to inconvenience or that they might be pricing out SCTs.

One interesting finding is the few patients reported ClinicalTrials.gov as a source of information for seeking SCTs. ClinicalTrials.gov has previously been cited as a potential area where clinics advertise SCTs as legitimate, pay-to-participate experimental research^31^. Our data suggests that perhaps policies to increase oversight of the ClinicalTrials.gov^32^ may not serve to better inform patients that some of the interventions listed in the registry are not actual research studies.

There are two limitations worth noting. The first is that patient self-recorded responses may not reflect the depth of factors and reasons for why they sought SCTs for their musculoskeletal condition. Additional quantitative and qualitative approaches would help triangulate and provide further evidence on which to corroborate or refute findings reported here. Our previous study showed that patients contacting the Regenerative Medicine Consult Service are information seekers and highly trust the institution^19^. Thus, a second limitation is that the patient population contacting our consult service and used in this study may not reflect the attitudes of the wider patient population interested in orthobiologic and SCTs for musculoskeletal conditions.

The responsible translation of orthobiologics involves good science, but also includes listening to patients and understanding their desires. Our findings from a large, prospective cohort of patients demonstrate that many patients considering orthobiologic and SCTs have undertaken prior online research and discussed them through a range of interpersonal connections, including providers at other clinics, before contacting our consultation service. Our results also indicate that patients express a diversity of reasons for wanting to undertake an orthobiologic or SCT. Some of these reasons focused on clinical evidence (e.g., treating or alleviating pain), while other reasons focused on intended outcomes (e.g., stem cells were better than other forms of care including surgery) were not informed by clinical evidence. While studies have shown the pervasiveness of online misinformation about SCTs, it remains unclear the extent to which misinformation may have influenced patient decisions amongst those in our cohort. These observations suggest that some patients may benefit from patient-centered counseling and consultation approaches that would serve increase health literacy but its impact on health behavior needs further investigation.

Physicians interested in the responsible translation of stem cell and orthobiologic therapies should make efforts to properly consult patients about the different options while maintaining a respectful understanding of the varied and complex motivations behind the reasons why patients seek such therapies. This may require additional time to counter preconceived beliefs that might be inaccurate and may present challenges to effectively correct. Physicians can use both informational and relational approaches to counsel patients^33^. They can provide fact-based information and alert patients to credible sources of information but be careful that in doing so does not discount or dismiss patients’ knowledge or their concerns over their illness^34^. Open dialog and empathic communication enhances the patient-physician relationship and provides an opportunity for patients to make informed decisions that are in their best health interests when considering experimental regenerative options for musculoskeletal conditions.

## Methods

All participants in this study expressed interest in “stem cell therapy” to treat an orthopedic condition, and while we tailor our educational content to differentiate between unproven stem cell interventions and more commonly used compliant orthobiologic therapies, patient use of the common term “stem cell” was still employed to communicate effectively in a clinical manner about certain procedures. Patients were either self-referred or seen in consultation at the request of another healthcare provider both inside or outside Mayo’s healthcare system as part of the Regenerative Medicine Consult Service initiated in 2016^19^. A patient outcomes registry was developed in 2018 to track validated orthopedic patient-reported outcome measures of every patient seen as part of the consult service or treated with an orthobiologic intervention^28^. As this was a retrospective review of patients’ medical information provided as part of routine clinical care, written consent was not obtained or required. This study was approved by the Mayo Clinic Institutional Review Board in accordance with institutional policies (IRB#: 20–003301).

### Data collection

Patients who contacted our appointment office directly or were referred by another medical specialty seeking SCTs were sent an electronic link to learn more about the consult service and to complete a questionnaire through a web browser or an iPad when attending in person. Included amongst the intake questions were three questions to capturing the perception and initial understanding of available SCT: (1) *Why are you interested in stem cell treatment for your condition?* (2) *How did you find out about stem cell treatment for your condition?* and (3) *Have you contacted another stem cell clinic to speak with them about stem cells?* These questions were chosen based upon the academic literature on patient perceptions surrounding stem cell interventions^18,21,22,25,35–37^ in order to help clinicians speak with patients about regenerative options to meet their needs. When formulating the questions, we allowed the term “stem cells” for the purpose of consultation even when discussing orthobiologic injections that contain no mesenchymal or other stem cells.

Patient questionnaires were collected between November 2018 and February 2020 utilizing an electronic software platform (Input Health, Vancouver, BC)^38^. Not all patients that completed questionnaires followed through with an in-person consultation, but their responses were included in the analysis. In addition to the responses to the three questions, patient demographics including age, gender, and whether or not they presented for in-person or virtual consultation was captured. Responses to questions 1 and 2 were entered as free text by patients with no word limit while a fixed response of Yes or No was given for question 3.

### Data analysis

We analyzed 533 patient surveys and the first 100 responses of questions 1 and 2 were used to develop the first draft of a codebook. Two coders (J.R.A. and S.A.S.) openly and independently coded responses into categories and then met to develop a codebook. Similar codes (response categories) were combined to create a final codebook of 16 possible responses to question 1 gauging interest in pursuing a SCT and 13 possible responses to question 2 to determine how the patient found out about a SCT.

Utilizing the final codebook, patient responses to the first two questions were categorized by the primary coder (J.R.A.). In some cases, patients gave multiple responses and those were categorized into more than one response category. For a randomly selected group of 50 patients, free-text responses were categorized by a second independent coder (S.A.S.) for use in inter-rater agreement analysis. Agreement between the original categorizations and the categorizations by the second independent coder were considered excellent and are summarized in Supplementary Table 1.

Common patient response categories were combined for statistical analysis purposes.

Additionally, we correlated the answers to the free-text questions with whether or not patients had previously contacted a stem cell clinic about treatment options as well as patients’ decision to present for face to face consultation as opposed to phone inquiry alone.

### Statistical analysis

Continuous variables were summarized with the sample median and range. Categorical variables were summarized with number and percentage of patients. Comparisons of reasons why patients reported that they were interested in a stem cell treatment for their condition according to (a) whether they had contacted another clinic to speak to them about a SCT for their condition, and (b) whether they eventually presented to Mayo Clinic’s Florida Campus for an in-person consultation were made using Fisher’s exact test. Comparisons of responses for how patients found out about a SCT for their condition, and also whether patients had contacted a clinic to speak to them about a SCT for their condition, and whether they presented for in-person consultation were also made using Fisher’s exact test. Agreement in free-text variable categorizations between the original classifications and those made by the second independent reviewer were assessed by estimating the percentage of agreement, and also by estimating kappa. *P* values < 0.05 were considered statistically significant and all statistical tests were two-sided. Statistical analyses were performed using R Statistical Software (version 3.6.2; R Foundation for Statistical Computing, Vienna, Austria).

### Reporting summary

Further information on research design is available in the Nature Research Reporting Summary linked to this article.

## Supplementary information

Supplementary Information

Reporting Summary

## Data Availability

The data that support the findings of this study are available from the corresponding author upon reasonable request.

## Code availability

The authors declare that the codebook description and final codebook to support the findings of this study are available in Supplementary Information files.
