# Author Correction: Patients seeking stem cell therapies—a prospective qualitative analysis from a Regenerative Medicine Consult Service

**DOI:** 10.1038/s41536-023-00278-3

**Published:** 2023-02-02

**Authors:** Jennifer R. Arthurs, Lisa M. Nordan, Brian H. Hultgren, Michael G. Heckman, Dayana Martinez, Zubin Master, Shane A. Shapiro

**Affiliations:** 1 Center for Regenerative Medicine, Mayo Clinic, Jacksonville, FL USA; 2 Robert D. and Patricia E. Kern Center for the Science of Health Care Delivery, Mayo Clinic, Jacksonville, FL USA; 3 Division of Biomedical Statistics and Informatics, Mayo Clinic, Jacksonville, FL USA; 4 United States Navy, Washington, DC USA; 5 Biomedical Ethics Research Program and the Center for Regenerative Medicine, Mayo Clinic, Rochester, MN USA; 6 Department of Orthopedic Surgery, Mayo Clinic, Jacksonville, FL USA; 7 Present Address: Center for Regenerative Medicine, Mayo Clinic, Jacksonville, FL USA

**Keywords:** Translational research, Medical ethics

Correction to: *npj Regenerative Medicine* 10.1038/s41536-022-00215-w, published online 25 March 2022

In this article, the affiliation ‘Center for Regenerative Medicine, Mayo Clinic, Jacksonville, FL, USA’ for Dayana Martinez was missing. The original article has been corrected.

