# Supplementary material for: Patients seeking stem cell therapies—a prospective qualitative analysis from a Regenerative Medicine Consult Service
Source: NPJ Regen Med. 2022 Mar 25;7:20. doi: 10.1038/s41536-022-00215-w (PMC8956610; doi:10.1038/s41536-022-00215-w)
Supplement: Supplementary file 1 — Supplementary Information [file 41536_2022_215_MOESM1_ESM.pdf]

Supplemental Content:

Codebook

Codebook Development

To analyze patient responses to the stem cell interest questions, a codebook was developed to identify major themes for two of the three patient answers. The third stem cell interest question was a yes or no answer that required no codebook. The codebook was developed after an initial review of the first one hundred patient responses to each of the relevant questions. Many of the patient responses fell into several subsets of responses which were then narrowed down to those listed in the codebook below. Qualitative answers were placed into the categories listed below using the codebook based on several "key words" identified by the reviewers. Based on the inductive coding, for each question, each patient response was categorized to fit into one or more of the following themes.

Question 1: Why are you interested in stem cell treatments for your condition?

We identified 14 thematic codes based on patient responses to question 1.

1. Treat or alleviate pain
Patient responses mentioned the goals of pain relief or treating pain.

2. Restore Function
Patient responses mention augmenting or improving function and may use keywords such as "function" or "range of motion." The description may include improving a physical or sporting activity.

3. Avoid/delay joint replacement or tendon repair
Patients described needing either joint or tendon surgery and reported that they wanted to avoid or delay surgery. Keywords used in this description may include "don't want surgery," or "delay surgery."

4. Medical contraindication to surgery
Patients listed an unrelated medical illness and had been told they had a medical contraindication to surgery or that surgery was not advised because of a medical comorbidity.

5. Less invasive than surgery, stem cells are better than surgery
Patients reported stem cell procedures were less invasive than surgery or explained that stem cells are "better" in some way to surgical options that may have been recommended for them.

6. Repair and or regenerate tissue
Patients described that stem cell treatments can be used to regenerate, replace, or repair cells, tissues or refer to those terms in the context of improving their condition.

7. Nothing Else Worked
Patients report having tried and failed other conservative treatments. They may or may not list previous treatments.

8. Natural Option
Some patients reported that because stem cell treatments use their own cells, that these procedures offer a more "natural" option than other conventional standard of care. Patients may use the word "natural."

9. Stem Cells are Better than Standard of Care
Patients reported by using terms like "better" or superior in reference to stem cell treatments compared to standard of care procedures. Patients reported specifically mentioning stem cells as an alternative to "drugs", did not want to take drugs and had to utilize the keyword "drug(s)", a specific medication or "medications."

10. Referred by a Healthcare Provider
Patient reported their interest in stem cell treatments was due to a recommendation from a healthcare professional. In some cases, the patient may provide the name or identify the healthcare provider who referred the patient. The healthcare provider may have been another physician, or physical therapist, etc.

11. Recommended by a Family or Friend
Patients reported that a family member or friend recommended or referred stem cells to them. In some cases, the patient may outline that the family member or friend benefited from the stem cell procedure.

12. Previously Had a Regenerative Treatment and Found it Helpful
Patients reported that they had previously tried a regenerative treatment (for the same or different medical condition) and found it helpful. A specific regenerative treatment may be mentioned including platelet-rich plasma bone marrow aspirate concentrate, or mention amniotic, umbilical or adipose/fat stem cells. In some cases, a generic use of the words "stem cells" may be used to describe the regenerative treatment.

13. Interested in Research
Patients described their interest in participating in research and may have used the keywords "research," "clinical trials," or "study."

14. Other or Unclear Response
Any other reasoning that did not fit into one of the previous categories or was not easily understood. Patients that stopped typing mid response such that the response was unclear would be categorized as unclear.

Question 2: How did you find out about a stem cell treatment for your condition?
We identified 10 thematic codes based on patient responses to question 2.

1. Online Search or Internet Research

Patients who mentioned keywords such as “online”, “research”,” internet”, “website”, “published”, “Google”, “articles”, “web”, and “studies”. Social media was excluded.

2. Mayo Clinic Media
Patients that mentioned Mayo Clinic platforms such as “Mayo Clinic online”, “Mayo magazine”, “Mayo website”, and “Mayo newsletter”.

3. Recommended by friend/family
Patients reported that a family member or friend recommended or referred stem cells to them. In some cases, the patient may outline that the family member or friend benefited from or recommended the stem cell procedure.

4. TV or Print Advertisement
Any patients who described learning about stem cell treatment through traditional advertisement. Must use the term “ad” or “advertisement”.

5. Social Media or Television story
Patients who mentioned various social media platforms. Also, media such as traditional “news” story, You Tube and “podcast” but not advertisements.

6. Stem cell Seminar or Clinic
Patients who learned about stem cells through a “seminar” or “local clinic.” Response implied that the patient visited a stem cell clinic or attended a seminar.

7. Healthcare Provider Referral or existing Mayo Clinic patient
Patient reported their interest in stem cell treatments was due to a recommendation from another healthcare provider. In some cases, the patient may provide the name or identify the healthcare provider who referred the respondent. Existing Mayo Clinic patients who reported “I am a Mayo Clinic patient” were included in this category.

8. Clinicaltrials.gov
Those patients who specifically mentioned searching Clinicaltrials.gov.

9. I am also scientist or healthcare provider
Patients that reported they were a scientist, researcher, or health care professional.

10. Other or Unclear
Any other reasoning that did not fit into one of the previous categories or was not easily understood. Patients that stopped typing mid response such that the response was unclear would be categorized as unclear.

Question 3: How did you find out about stem cell treatment for your condition?
This question had a fixed response of “yes” or “no.”

Supplemental Table 1: Agreement between original categorizations and those of an independent reviewer for 50 randomly selected study subjects

Variable	Fraction (%) of agreement	Kappa
Reason why interested in a stem cell treatment		
Treat or alleviate pain	47/50 (94.0%)	0.86
Restore function	49/50 (98.0%)	0.66
Avoid/delay joint replacement or tendon repair	46/50 (92.0%)	0.81
Medical contraindication to surgery	50/50 (100.0%)	1.00
Less invasive than surgery, stem cell are better than surgery	47/50 (94.0%)	0.63
Repair, regenerate tissue	48/50 (96.0%)	0.87
Try alternative option, nothing else worked	49/50 (98.0%)	0.88
Try natural option	50/50 (100.0%)	N/A[1]
Stem cells are helpful/beneficial or better than standard of care	46/50 (92.0%)	0.30
Referred by a healthcare provider	50/50 (100.0%)	1.00
Referred by friend/family or friend/family had benefit from treatment	50/50 (100.0%)	1.00
Previously had regenerative therapy and found it to be helpful	50/50 (100.0%)	N/A[1]
Interested in research	50/50 (100.0%)	N/A[1]
Instead of drugs	50/50 (100.0%)	N/A[1]
Other	44/50 (88.0%)	0.36
How the patient found out about a stem cell treatment for their condition		
Online research	44/50 (88.0%)	0.76
Mayo Clinic Media	49/50 (98.0%)	0.79
Recommended by friend/family	49/50 (98.0%)	0.88
TV or Print Ad	49/50 (98.0%)	0.79
Social Media or TV Story	48/50 (96.0%)	0.00
Stem cell seminar or clinic	49/50 (98.0%)	0.88
Provider referral by healthcare provider or existing Mayo patient	50/50 (100.0%)	1.00
Clinicaltrials.gov	49/50 (98.0%)	0.00
I am also scientist or healthcare provider	50/50 (100.0%)	N/A[1]
Other	50/50 (100.0%)	N/A[1]

[1] It is not possible to estimate Kappa when there are not any patients in one of the two categories.

Supplemental Table 2. Example Quotes to Thematic codes in Responses to Questions

Thematic code	Example Quote
**Q1. Why are you interested in a stem cell treatment for your condition?**	
1. Treat or alleviate pain	To eliminate or reduce chronic shoulder pain
2. Restore Function	Knee stiffness limits some of my activities and quality of life
3. Avoid/delay joint replacement or tendon repair	I would like to explore this opportunity prior to considering knee replacement surgery.
4. Medical contraindication to surgery	I am unable to have replacement and heard this procedure has been successful.
5. Less invasive than surgery, stem cells are better than surgery	I believe it has great potential as a non-invasive way to cure, not just treat my condition.
6. Repair, regenerative tissue	I am interested in re-growing the cartilage tissue in my knee.
7. Try alternative option, nothing else worked	I have tried everything else and still have pain.
8. Try natural option	More natural than invasive procedures.
9. Stem cells are helpful/beneficial or better than standard of care	Reports are positive for the stem cell treatment with minimum pain and side effects.
Instead of Drugs	To reduce knee and shoulder pain without narcotics.
10. Referred by a healthcare provider	My primary care doctor suggested I look into it as an option for knee replacement surgery.
11. Referred by friend/family or friend/family that had benefited from treatment	My husband has been successfully treated twice with stem cells.
12. Previously had regenerative therapy and found it to be helpful	Have had stem cell treatments 4 years ago. Positive effects have worn out.
13. Interested in research	I feel I would be a good candidate for the research for my hip and knee.
14. Other or Unclear	I would be interested to see if stem cells are an option to treat my condition.
**Q2. How did you find out about a stem cell treatment for your condition?**	
1. Online Research	I have been researching online and reading articles about this treatment.
2. Mayo Clinic Media	read about in a Mayo newsletter
3. Recommended by friends/family	recommendations from friends who had favorable outcomes of the treatment
4. TV or Print Advertisement	saw an ad in the newspaper
5. Social Media or TV Story	Joe Rogan podcast
6. Stem cell seminar or clinic	went to a seminar about what can stem cell treatment can do for you
7. Provider referral by healthcare provider or existing Mayo patient	referral from my orthopedic surgeon at Mayo Jacksonville

8. Clinicaltrials.gov	clinicaltrials.gov website
9. I am also a scientist or healthcare provider	as a PhD psychologist I am well read and well published
10. Other or	I was involved in a previous study 3 years ago
11. Unclear	I hope Mayo can help
